# Common Bias and Challenges in Physical and Rehabilitation Medicine Research: How to Tackle Them

**DOI:** 10.3389/fresc.2022.873241

**Published:** 2022-06-13

**Authors:** Aurore Thibaut, Charlotte Beaudart, Géraldine Martens, Stephen Bornheim, Jean-François Kaux

**Affiliations:** ^1^Coma Science Group, GIGA Consciousness, University of Liège, Liège, Belgium; ^2^Center du Cerveau 2, University Hospital of Liège, Liège, Belgium; ^3^Department of Rehabilitation and Sports Sciences, University of Liège, Liège, Belgium; ^4^World Health Organization Collaborating Center for Public Health Aspects of Musculoskeletal Health and Aging, Department of Public Health, Epidemiology and Health Economics, University of Liège, Liège, Belgium; ^5^Réseau Francophone Olympique de la Recherche en Médecine du Sport (ReFORM) International Olympic Committee (IOC) Research Center for Prevention of Injury and Protection of Athlete Health, Liège, Belgium; ^6^Department of Physical Medicine and Sports Traumatology, Sports, FIFA Medical Center of Excellence, FIMS Collaborative Center of Sports Medicine, University and University Hospital of Liège, Liège, Belgium

**Keywords:** clinical trial, evidence-based medicine (EBM), treatment, traumatology, study design

## Abstract

The importance of evidence-based medicine is crucial, especially in physical and rehabilitation medicine (PRM), where there is a need to conduct rigorous experimental protocols, as in any medical field. Currently, in clinical practice, therapeutic approaches are often based on empirical data rather than evidence-based medicine. However, the field of PRM faces several challenges that may complicate scientific research. In addition, there is often a lack of appropriate research training in educational programs. In this context, we aim to review the methodological challenges in PRM and provide clear examples for each of them as well as potential solutions when possible. This article will cover the following themes: (1) Choosing the right study design and conducting randomized and benchmarking controlled trials; (2). Selecting the appropriate controlled, placebo or sham condition and the issue of blinding in non-pharmacological trials; (3) The impact of populations' heterogeneity and multi-comorbidities; (4). The challenge of recruitment and adherence; (5). The importance of homogeneity and proper quantification of rehabilitative strategies; and (6). Ethical issues. We are convinced that teaching the basics of scientific research in PRM could help physicians and therapists to choose a treatment based on (novel) scientific evidence. It may also promote scientific research in PRM to develop novel and personalized rehabilitation strategies using rigorous methodologies and randomized or benchmarking controlled trials in order to improve patients' management.

## Introduction

Most of the current research in physical and rehabilitation medicine (PRM) is built on applied research, which by definition is research that uses existing knowledge to achieve specific goals; they are designed to solve a specific problem affecting a specific individual or a group of patients. The field of research in PRM is highly translational (1), meaning that the aim is to transfer data obtained from scientific research (e.g., in laboratories) into clinical setting results (e.g., in rehabilitation), commonly referred to as the “Bench to Bedside” approach. However, conducting translational research is extremely complex as it encompasses many biases and risks that must be considered in order to provide robust results that can be replicated and recognized as newly validated rehabilitation strategies. Indeed, even if a lot of progress has been made to implement evidence based medicine in clinical practice, this transition should be further accelerated (2).

As any medical fields, the development and progress of novel and optimized interventions in PRM depends on their validation in well-designed research protocols. However, due to several factors (e.g., concomitant therapies, blinding difficulties, proper controlled condition, patients' heterogeneity), the implementation of double-blind randomized placebo controlled trials (i.e., the gold standard in evidence based medicine), can be extremely complicated depending on the intervention studied. Indeed, rehabilitation, in most cases, encompasses very heterogeneous inter and intra-individual approaches, which is in contradiction with the homogeneity and standardization of interventions required in research protocols. In addition, as compared to strict research protocols, where the goal is to control the patient's external environment, the final objective of research in PRM is to enhance the effectiveness of an intervention in real world circumstances (3, 4). In this context, the notion of real-effectiveness medicine (REM) has been introduced by Malmivaara and proposes a certain balance between the necessary robustness of research protocols and the real-life constraints of PRM (3). REM recommends to act on different levels as follows: (1) benchmarking (i.e., learning from the peers); (2) quality (i.e., real world performance); (3) evidence-based medicine (EBM; i.e., up-to-date scientific evidence) and (4) competence (i.e., basis for effectiveness, efficacy and equity). This framework has been proposed to provide the best cares for patients in real-world settings (as opposed to research settings).

In parallel with this, recently, the Cochrane Rehabilitation experts consortium have proposed the following definition for rehabilitation in the context of research: “*In a health care context*, rehabilitation is defined as a “*multimodal, person-centered, collaborative process*” (**Intervention-general**) including interventions targeting a person's “*capacity (by addressing body structures, functions, and activities/participation) and/or contextual factors related to performance*” (**Intervention-specific**) with the goal of “*optimizing*” the “*functioning*” (**outcome**) of “*persons with health conditions currently experiencing disability or likely to experience disability, or persons with disability*” (**Population**) (5). As stated by the authors, this definition has the advantage of providing explicit inclusion and exclusion criteria; could impact future research production; is a first edition, thus may be revised in the future (5).

As more and more (Ph.D) students and clinicians in PRM are involved in research, it is critical to provide them with the appropriate tools to successfully and efficiently carry out research projects. In this context, this article aims to discuss critical aspects to take into account when testing the effect of an intervention in the context of PRM, in order to promote robust research in this field and enhance the clinical translation of evidence based practice.

## Selecting the Appropriate Study Design

Scientific research should always begin with the development of a research question. For each research question, it is important to identify the appropriate research design that could answer it. In general, there are three main study designs: *descriptive, exploratory* and *experimental*. A descriptive design will be used when the objective is simply to describe a particular population (e.g., describe the intensity of pain or the mobility capacities of a population suffering from low back pain); an exploratory/analytic design will be used when the objective is to describe a relationship between two variables (e.g., investigate the relationship between lower limb amputation and quality of life or investigate if age can be a predictive factor of the recovery of lower back pain); an experimental design will be used when the objective of the study is to investigate the effect of a rehabilitation protocol on a particular population (e.g., efficacy of hippotherapy vs. usual care on motor capacities of patients suffering from multiple sclerosis - MS).

Epidemiological studies using descriptive or exploratory designs are classified as “observational studies” or “non-experimental studies”. No intervention is used, and no attempt to alter the course of the disease is made. Different observational studies exist as exposed in Table 1.

**Table 1:** Different observational study designs.

**Cross-sectional studies – *descriptive design* (6, 7)**
A cross sectional study involves looking at data from a population at one specific time point. It can be considered as a snapshot of a particular population at a particular point of time. These studies are often used to look at the prevalence of some characteristics in a given population (e.g., prevalence of depression in a population of patients suffering from MS). This type of study can be used to “describe” characteristics that exist in a population but not to determine cause-effect relationship between variables (e.g., it cannot determine if MS leads to higher depression symptoms). Cross-sectional studies can be used to gather preliminary data to support further research.
**Case-control studies** ***– exploratory design*** (8, 9)
A case-control study compares patients with a certain disease (or an outcome of interest), i.e., the “cases”, with patients who do not have the disease, i.e., the “controls” and looks at how frequently both groups have been exposed to a risk factor. If more participants in the case group experienced the risk factor, this suggests that it is likely that there is a link between the risk factor and the disease. These studies are usually retrospective, appropriate for studying rare conditions or rare diseases, often easy to implement and do not require a lot of time and they give the opportunity to simultaneously look at multiple risk factors. However, they are often confronted with low data quality because they rely on retrospective data, and sometimes, recall bias. Recall bias in a case-control study is the increased likelihood that those with the outcome (e.g suffering from a disease) will recall more and report exposures (e.g. symptoms) compared to those without the outcome. This may lead to concluding that there are associations between the exposure and the disease that do not, in fact, exist. Moreover, due to their typically retrospective nature, case-control studies can be used to establish a correlation between exposures and outcomes but cannot establish causation. These studies simply attempt to find correlations between past events and the current status of a condition.
**Cohort studies (prospective cohort studies or retrospective cohort studies) –** ***exploratory design*** (10, 11)
A cohort study is a longitudinal study were participants, who usually share a common characteristic, are followed over a certain period of time to evaluate the occurrence of an outcome of interest. A correlation between the exposure to risk factors (that can be present or not at the beginning of the follow-up) and the development of a particular outcome is measured. (e.g., follow-up of patients suffering from spinal cord injury and evaluation of the occurrence of different outcomes such as hospitalization and mortality between different types of spinal cord injuries). Cohort studies are very important in epidemiology to understand which risk factors may increase or decrease the likelihood of developing an outcome or a disease. Cohort studies may be prospective or retrospective. In prospective cohort studies, when a population is included in the study, the potential exposure of interest is first measured. Then, the population is classified as having been exposed or not exposed to the risk factors and participants are followed prospectively over time. The investigators then assess the outcome of interest in these individuals. In retrospective cohort studies, data is collected from records; the outcomes have occurred in the past. Cohort studies are therefore robust and effective to establish cause and effects, which cannot be established with the previous study designs. A cohort study may be helpful to study multiple outcomes to the same exposure but also helpful to study the relationship between outcome and exposure, even if the exposure is rare. However, collecting prospective data from a large number of patients over many years is often challenging, time-consuming and expensive. For example, to study the incidence of cardiovascular events in patients suffering from Parkinson, researchers may have to follow the cohort for many years before the studied outcome occurs. Indeed, cohort studies may not be very efficient for rare outcomes (for which a case-control study may be preferred), except in some conditions.

Studies using an *experimental design* are classified as “*experimental studies*”. In a clinical trial, study participants are usually divided into two groups; one group receives an intervention and the other group does not. An outcome of interest is then compared between these groups and estimates the impact of the intervention. The clinical trials may be classified into different types (12, 13), namely, parallel, crossover and factorial designs (see Table 2), depending on the objective of the study and the population studied.

**Table 2:** Different interventional study designs.

**Parallel group design**
Parallel group design is the most common form of clinical trials. In these trials, participants are divided into two (or more) groups, with one of the groups receiving the intervention and the other not (i.e., controlled condition – see section Selecting the Appropriate Study Design for more details).
**Cross-over design**
In a cross-over design, each patient receives both interventions but in a different, randomized, order. Half of the participants therefore start with treatment A and then switch to treatment B (AB sequence). The other half of the participants start with treatment B and then switch to treatment A (BA sequence). An adequate washout period should be considered in order to eliminate the effect of the previous intervention before starting the second one, to avoid a carry-over effect. In this case, each participant serves as his/her own control. This type of study therefore requires a smaller sample size, which is a significant advantage in PRM. It is important that the condition is chronic, relatively stable and does not get completely cured during the first part of the trial. One disadvantage of this study design is that the duration of follow-up for the patient is longer than the duration for a parallel group design, which increases the risk of dropout and increases the risk of leading to a compromised study power. In addition, cross-over designs are not suited to investigate multiple dose levels of an intervention.
**Factorial design**
In factorial design experimental studies, the investigators tests more than one intervention simultaneously (e.g., multimodal interventions such as a combination of dietary supplements and physical rehabilitation for the improvement of muscle strength in patients with transfemoral amputation). This study design is appropriate for the study of two or more interventions using various combinations. Often, 4 groups of participants are included; the first one receives both interventions, the second group receives the first intervention, the third group receives the second intervention, and the last group serves as control group. This study design is very helpful because both interventions may be tested at the same time. Moreover, sample size requirements are often lower. However, recruitment for these studies is challenging since participants should meet inclusion criteria, not only for one intervention but for two or more at the same time. Moreover, these studies also require an absence of interaction between interventions.
**Superiority trials**
In *superiority trials*, the purpose is to assess if one intervention is different compared with another intervention or with a placebo. The null hypothesis is that there is no difference between groups and the alternative hypothesis is that there is a difference between both interventions.
**Equivalence trials**
In *equivalence trials*, the purpose is to show that a new intervention is equivalent to another existing one. Certain advantages of the new intervention may explain researchers interest in it, such as better safety or higher cost-effectiveness. In these studies, the null hypothesis is that the difference between the new intervention and the standard intervention is greater than a certain predefined effect size and the alternative hypothesis is that the difference between the tested intervention and the standard intervention is not greater that this predefined effect size.
**Non-inferiority trials**
In *non-inferiority* trials, the purpose is to show that a new intervention is “not worse” compared to another existing intervention in terms of efficacy, but demonstrates, for example better safety issue. In such a trial, the null hypothesis is that the difference between the new intervention and the existing one is greater that a certain predefined effect size and the alternative hypothesis is that the difference between both intervention is lower than this predefined effect size.

In addition, clinical trials may also be categorized according to their purpose. We can differentiate *superiority trials, equivalence trials* and *non-inferiority trials* (12).

Even though all study designs can be used in PRM, they do not have the same level of evidence. The evidence-based medicine pyramid can be used to rank these studies according to their level of evidence. Studies at the top of the pyramid are studies with a higher level while studies at the bottom of the pyramid are studies with a lower level of evidence. The main reason is that the higher we go in the pyramid of evidence, the lower risk of methodological bias there is.

In PRM, although placebo randomized controlled clinical trial are not always possible to implement because of different methodological problems (14) (Table 3), these studies remain the gold standard design. Many interventions may be non-pharmacological interventions (e.g., injections, rehabilitation protocols, noninvasive brain stimulation, aqua therapy, acupuncture, etc.) which reinforce the difficulties inherent to the implementation of such study design.

**Table 3:** Methodological problems in rehabilitation research (14).

**Categories of issues**	**Type of issue**
***Control group***	Difficulties in having a “placebo” group. For some interventions, it may be possible to reproduce the non-pharmacological intervention using a placebo device but, most of the time, the control group will receive no intervention. The placebo effect of the intervention could therefore not be assessed.
***Blinding***	Difficulties in blinding participants and personnel. Indeed, participants are aware of their group of appurtenances since, they will either receive the intervention or not. For the staff, it is a challenge to ensure that the person applying the treatment is not the same as the person carrying out the measurements.
***Randomization***	Limited participant's acceptance of randomization. Participants are often hesitant of being randomly assigned to one group and, particularly to the control group with no intervention offered. Patients are often in pain or suffer from important mobility issues/disabilities. Therefore, when they are included in the control group, they only need to perform the measurement at the entry and at the end of the study and they do not perceive any benefit from participating in the study.
***Ethics***	Unacceptability to use a control group that withholds or delays treatment. Patients assigned to the control group often receive no intervention and are required to not modify their current rehabilitation.
***Eligibility***	Existence of multiple comorbidities that restrict the inclusion of participants. Therefore, there is an insufficiency of eligible participants in one unique site of recruitment and multisite studies often have to be organized. Difficulties to recruit participants with pathologies that can be considered as similar on the phenotypic level.
***Monitoring of interventions***	Complexity of some interventions that makes it difficult to monitor their administration.
***Confounding factors***	The multifactorial rehabilitation of PRM patients that makes difficult to identify the true intervention effect and differentiate the aspects of natural recovery processes within the course of their rehabilitation.
***Participant attrition***	Interventions proposed in PRM research may be long in term of follow-up, which increases the risk of participant attrition.

Because of the difficulties related to the implementation of randomized controlled trial (RCT) in PRM research, one-way design studies, called pre-posttest studies are sometimes conducted, as an alternative. In these studies, only one group of patients is included and receives an intervention. To show the effect of the intervention on a particular outcome, the independent variable is measured before and after the intervention. These studies are the weakest type of experimental design. A major limitation is the lack of a comparison group and the impossibility to measure truly the impact of the intervention, given the large amount of cofounding factors that may be present in PRM patients.

Another, alternative to placebo randomized controlled clinical trial are *pragmatic trial* (15). Pragmatic trials are designed to evaluate the effectiveness in real-life routine practice conditions and produces, therefore, results that can be applied in routine practice clinical settings. Contrarily to experimental designs such as placebo randomized controlled trials that aims to test whether an intervention works under optimal conditions (e.g., inclusion/exclusion criteria, standardized protocols regarding the intervention, etc.), pragmatic trials are conducted in real-world clinical practice settings, with typical patients and by qualified clinicians. In pragmatic trials, inclusion criteria are often less strict, randomization could be performed at the group level (e.g., one group of participants could be treated in a particular setting and a second group of participants, matched to be similar to the first group might serve as the control group) and a wide spectrum of outcomes, mostly patient-centered, could be considered. Because they better suit with research in PRM, pragmatic trials are becoming increasingly popular in this area of research. Besides, the notion of benchmarking controlled trial (BCT) (observational study) has been recently proposed as an alternative to RCT (experimental study) as it might be more suitable for translational research especially in the field of PRM (16). In addition, in another opinion paper, the author exposed the notion of clinical impact research and the necessity to conduct more BCTs as this study designs are closer to the real-world constrains in rehabilitation, advocating that the translation of the results from RCT are limited (17, 18). The term benchmarking is derived from the necessity to make between-peer comparisons, and thus learn from the best practices. In short, BCT aims at assessing the efficacy of one or multiple interventions or clinical pathways, in real-world settings, thus being observational. BCT might be preferred to RCT due to ethical considerations or feasibility reasons, as well as when it comes to the evaluation of the efficacy of a clinical pathway or performance of health care providers (17). Specific recommendations have been proposed to develop and evaluate the effectiveness of observational BCTs. In the future, more BCTs might be available in the literature [for more information on this topic see (16–19)].

One important aspect when it comes to conduct a study protocol, is the reporting guidelines. Such Guidelines have been developed to help researchers report all details of their study allowing for an appropriate understanding by readers, a proper replication of the study protocol, Its use by a clinician for a clinical decision as well as to be included in a meta-research study. In 2015, EQUATOR, created a flow chart to help authors identify the most appropriate guidelines based on their study design. The EQUATOR website (https://www.equator-network.org/reporting-guidelines/) provides a census of all the available reporting guidelines. In short, for observational studies, the STROBE checklist and extensions may be used and for clinical trials, the CONSORT checklist and extensions (e.g., extensions for cross-over trials, non-inferiority trials, etc.) may be used.

Recently, Cochrane Rehabilitation Methodology Meetings have been organized with the aim to improve the methodology used in PMR to generate effective and translational evidence (20). In this context, the RCT Rehabilitation Checklists (RCTRACK) project have been proposed to develop specific guidelines specific for research conducted in rehabilitation (20), whose work is ongoing.

## Choosing a Proper Controlled Condition and Blinding Strategies

As described in the previous section, RCTs are the gold standard to test the efficacy of a novel treatment. Nevertheless, compared to pharmacological RCTs, finding an appropriate controlled condition (i.e., placebo) might be extremely challenging in the context of PRM, which makes the design of RCTs complex. Placebo controlled double-blind trials are however crucial to validate the efficacy of a treatment as uncontrolled and open-label trials suffer from important risk of biases that can alter the validity of an observed finding. However, it should be noted that blinding is not always warranted in rehabilitation (21). For instance, when the study question is about effectiveness in routine health care, blinding might be questionable. In addition, when the study question is about effectiveness in routine health care, blinding is also questionable. Indeed, when it comes to translating the studied intervention to the real clinical world, the patient will be aware of the treatment he/she will receive, thus a combination of the treatment effect *per se* and a certain degree of placebo effect will occur (21).

In the context of PRM research, there are two important risks of bias. Performance bias corresponds to (behavioral) changes that occur due to the knowledge of interventions allocation, from either the researcher or the participant or both. Indeed, a patient who knows he/she is receiving a new therapeutic intervention is more likely to experience a placebo effect (i.e., clinical improvement in the absence of any ‘true' intervention). This placebo effect will bias the results as it will be impossible to disentangle the treatment effect (due to the intervention) from the improvement due to the patient's expectation (i.e., placebo effect). In addition, the assessors, who are not blinded of the treatment allocation, might be influenced as well and prone to oversee clinical enhancements. Finally, the care-givers might also be biased, and subconsciously provide care that differs between the treated and the control group. This bias can be prevented by using appropriate blinding for both the participants and the researcher or the clinician providing the treatment. It can also be minimized by using adequate placebos (22); however, as said previously, it is not particularly simple to develop double-blind placebo controlled trials in PRM.

The other main bias is detection bias, which is defined as systematic differences between groups in how the outcomes are determined, which can cause an overestimation or, on the other hand, an underestimation of the size of the effect. Such bias can easily be prevented using randomization procedures to ensure that the two groups (active and placebo) are homogenous and comparable.

Many trials conducted in PRM are open-label studies (i.e., the researchers and participants know which therapy is being administered), which may induce an important placebo effect both for the patients and the researcher, thus inducing important biases on the outcomes (23). Such open-label designs, even if they can bring some important insights on the possible efficacy of an intervention as well as its safety and feasibility, do not allow for any conclusion to be drawn regarding its actual efficacy.

Beside open-label studies, in PRM, most clinical trials either use no intervention, standard care or other conventional interventions as a control group. These (non-)interventions might not only be problematic in term of the amount of treatment received (the intervention group will receive more therapy than control group), but make blinding impossible and randomization complicated. Therefore, the development of appropriate placebo or sham procedures is crucial. One possibility to develop valid controlled conditions in trials assessing active rehabilitation strategies (e.g., the effect of a robotic rehabilitation on muscular strength in patients with MS) would be to apply another *active* intervention not expected to cause any effect (e.g., robotic rehabilitation using movements too slow to impact muscular strength, the use of aerobatic vs. anaerobic exercises as a controlled condition). In this context, a collaboration with companies to develop appropriate placebo devices and protocols would be beneficial [for instance, a shockwaves head was manipulated by the company to mimic but not delivering shockwaves and unsure a proper blinding (24)]. Nevertheless, if such approaches might be enough to ensure a proper blinding of the patients, it might fail to properly blind the therapist.

To add to these challenges, rehabilitation strategies are highly heterogeneous both at the intra and inter individual levels. Indeed, based on the patient's level of impairment (i.e., rehabilitation based on deficits), the techniques and amount of therapy a patient will receive can vary for the same pathology. In addition, for the same patient, depending on other clinical factors such as fatigue, emotional status or concomitant treatment, sessions of therapy can greatly differ from 1 day to another. Of note, in other fields, such as neuromodulation and non-invasive brain stimulation, personalized approaches using for instance, electrode montages based on the patients' individual brain lesions, are being developed, which will greatly reduce the homogeneity of the intervention provided. While such heterogeneity might be seen as a limitation in research, it is crucial for patients' management. One solution to be able to quantify the amount of therapy provided could be to monitor the time and the load for the patient using the Borg scale, the rating scale of perceived exertion (25). These parameters could then be taken into account in the statistical analyses. Additional parameters (e.g., number of repetitions, distance covered, strength) could also be considered based on the intervention.

In addition, the heterogeneity of the intervention is also a challenge given the heterogeneity of the disease or studied population (see next section). In this context, models have been proposed, such as the FITT model for exercise (26), accounting for the multidisciplinary intensity of care based on the Frequency, Intensity, Time, and Type of exercise proposed. Such program may offer more flexibility to the proposed rehabilitation program based on specific guideline, allowing to quantify the amount of exercise performed by patients.

In some cases, having a controlled group is impossible. For instance, when it comes to testing the efficacy of a prosthesis, it is almost impossible as this would require the development of a ‘sham' prosthesis. This is not only extremely costly but the setting of appropriate and true sham parameters is almost impossible to achieve. In addition, it might be ethically questionable not to offer a potentially beneficial treatment to half of the participants. To circumvent this issue, one could offer the treatment to the control group after the study completion. Another challenge in PRM, is that placebo treatments need to be efficient for blinding over longer periods of time, as most therapies are provided for several weeks or even months. This makes the blinding and masking aspects even more challenging.

Even in controlled studies, successful blinding is often hard to achieve in PRM (22, 27). In a systematic review evaluating the amount of RCTs correctly reporting blinding strategies, the authors found that an important majority of trials do not correctly report it, but most importantly, that trials with positive results tend to have lower reporting rates for correctly reporting blinding (28). The fact that unblinded trials report more positive results compared to blinded studies is well known, not only in the field of PRM. Therefore, more effort is required to develop reliable blinding strategies and clearly report them in study protocols. One simple but costly solution is to have two clinicians or researchers involved in the protocol, one responsible of applying the intervention (active or placebo/sham) and the other one in charge of the assessments. This also implies that the patient has to receive the study interventions in a separate room (as opposed to conventional physical treatment often performed in large rooms where several patients receive their care simultaneously). Even if considered simple, this strategy might be complicated to implement as it requires additional financial resources.

## The Challenge of Populations' Heterogeneity

To add to these existing methodological challenges, the population targeted in PRM research is often heterogeneous in terms of symptomatology, even within a same pathology. Traumatic brain injury (TBI), for instance, encompasses a wide variety of clinical symptoms ranging from mild (e.g., troubles to concentrate, headaches) to moderate (e.g., impaired executive functions, ataxia) and severe (e.g., disorders of consciousness, paresis), while it is often studied in interventional studies (e.g., cognitive-behavioral interventions, non-invasive brain stimulation). TBI is therefore complex to recruit a homogeneous study sample. One solution is to apply stringent inclusion criteria in terms of symptoms however this does not only compromise the recruitment; it also decreases the external validity of the study. The clinical translation of a therapeutic method tested on a highly selected population is indeed poor.

This heterogeneity combined with the aforementioned difficulties in designing methodologically robust studies and the recruitment issues, leads to small sample sizes and many studies are thereby statistically underpowered. The lack of standardization of the outcomes further makes the comparison between studies complex. All these factors lead to a poor representation of these studies in meta-analyses. The same applies to stroke patients where the heterogeneity of the populations in terms of type of stroke, clinical presentation and duration of the symptoms, makes the integration in meta-analyses complicated, despite an important amount of published studies.

Another factor specific to PRM (but also other fields such as cancer research) is the evolving aspect of some diseases. Indeed, neurodegenerative diseases such as MS or Alzheimer can have varying patterns of evolution with, for instance, important decreases in function over a short period of time followed by longer periods of disease stability. These evolutionary patterns can be very different from one patient to another and can further compromise the homogeneity of study populations, within a same group (e.g., experimental group) or between groups (experimental and control).

Finally, another contributing factor to the population's heterogeneity is the presence of comorbidities. Potential study candidates often suffer from other pathologies than the targeted one (e.g., diabetes, hypertension, arthrosis) and these varying profiles are very difficult to control for. It is however important to take these comorbidities into account notably because some of them may be risk factors for the targeted condition (e.g., diabetes is a risk factor for chronic tendinopathy).

All these aspects may be seen as barriers for conducting robust research in PRM and discourage researchers. However, there are several ways to tackle them, pending sometimes a shift in clinical and scientific routines (29). First, there is a consensus that larger samples have to be included. To do so, the field has to move from a segregated model to an open and collaborative network. Large multicenter trials, open science practices and registration of protocols are important and efficient paths to follow. Second, the current state of reporting regarding baseline patients' medical condition is still too low but could easily be improved based on standardized reporting guidelines and Common Data Elements [e.g., (30, 31)]. Finally, while heterogeneity can be perceived as a limitation from a purely methodological standpoint, it corresponds to the clinical reality and could be embraced as such. Pending sufficient sample sizes, analytical approaches accounting for the populations' heterogeneity can be used: clustering methods, normative modeling, and measures of individual changes, for instance.

Since there is no way of getting completely rid of the heterogeneity in populations for PRM research and given the high propensity of this field for individualized treatment approaches, measures should be taken to account for it and the study design, and the analyses should be planned accordingly. This can only be managed through a collaborative and inter-disciplinary approach.

In addition, a proper reporting of the population is crucial both for the generalizability of the findings of RCTs (32) and for consequent systematic review (33). So far, the percentage of adequate reporting is poor, thus limiting the generalizability of RCTs' results in clinical settings (i.e., reduced effectiveness). Future clinical trials should provide a clear description of the patients' selection and the study setting, as well as clear characteristics of the studied population in term of patients' functioning, comorbidities, as well as behavioral, environment and inequity factors (32).

## Improving Recruitment and Adherence

### Recruitment

Recruitment for PRM research, just as for most other research topics, can often be challenging. Clinical studies in this domain focus frequently on patients, each with their own life prior to the injury, and that continues despite it. This is different to studies done on a cellular level, or on animal models, as there is a high variability in behavior.

Research in this domain must also take into account a large number of variables that are a necessity for patients to continue functioning, despite their injury. For example, there are ethical implications, as well as research implications, by giving pain killers to subjects implicated in pain-management studies.

Difficulties encountered are also linked to the large number of monocentric studies run concomitantly. Patients that have very few co-morbidities, and who are ideal candidates, are sought after, making recruitment all the more difficult, the more studies are simultaneously run. As there is a large interest in running these studies in localized, well controlled environments, such as rehabilitation centers, or retirement homes, the population is often limited to the capacity of the centers, making recruitment more challenging. As stated above, running larger, multicenter studies, despite the logistical challenges, could help counteract this limitation.

There are also time constraints that can limit patients' willingness to participate in studies, in addition to their rehabilitation. There seems to be relatively little free time in between therapy, care, needed rest, functional daily live activities and socialization requirements (34, 35). It stands to reason that including novel or innovative technologies might increase recruitment, out of curiosity, personal beliefs, or lack of alternative solutions (36–38). Unfortunately, this doesn't always hold true (39), as new technology has its downfalls (ie: complexity of use, especially with an older population), though specifically targeted models might inverse this trend (40). Another setback is the difficulty in implementing a placebo treatment with these new technologies.

Patients might also simply lack awareness of clinical trials. Certain rehabilitation centers, associated with universities or research labs, have scientific coordinators, to help manage multiple studies that take place simultaneously, but it is usually the healthcare providers that recruit patients for studies (41).

### Adherence

Adherence is also a challenge for longitudinal studies. Motivation, beliefs, but also factors linked to the rehabilitation program (fatigue, discomfort, time constraints etc…) can increase risks of dropouts. As recovery tends to slow down, and results are sub-satisfactory, adherence can become challenging. There are steps that therapists and researchers can take to try and convince patients to stay the course such as explaining the benefits of the new technique, and setting obtainable goals with the patient instead of for the patient (42).

A large proportion of patients requiring rehabilitation are elderly. One of the leading factors influencing response rate (i.e., nonparticipation or lack of adherence) is age (though the data is controversial on exactly how age influences adherence (though the trends seem to point toward lower adherence during midlife (other occupations that take precedent), and with elderly subjects (physical or cognitive disabilities that hinder adherence (43). Other factors that negatively affect response rates are smoking, educational status and income which are also linked to higher disabilities levels for a variety of illnesses, such as arthritis (44), stroke (45), or lower back pain (46).

In the case of athletes, when rehabilitation is investigated, there seems to be similar adherence problems, with a very high percentage of participants reporting low adherence. This usually has to do with the very busy timetables, and many athletes want a “quick fix” (and therefor dropout relatively quickly). However, in a small proportion, some participants report over-adherence (where subjects overwork, or over train, which could lead to further injuries) (47).

Adherence depends on a variety of factors such as intrinsic motivation, speed of results, and the hassles linked to rehabilitation. These can be improved if medical professionals continue to motivate patients (48) through education and explanations.

Patient adherence to rehabilitation, and strategies to improve it, were recently put to the test. With the lockdown following the COVID-19 pandemic, patients needing rehabilitation found themselves isolated and unable to receive care. Telerehabilitation has been shown to be a promising alternative to face-to-face rehabilitation during the pandemic (49, 50).

Going back to the notion of BCTs vs. RCTs, patients' adherence might be limited in the context of RCT which required a strict compliance to the study protocol, thus increasing the attrition rate. On the other hand, as regard to effectiveness research, BCT might be preferred, limiting the risk of drop-out as study criteria represent the real clinical setting (51). On the other hand, the difference in baseline between the studied groups in BCTs should be taken into account as this can seriously influence the study outcome. However, lack of adherence in RCTs is an important limitation to the generalizability of their findings.

### Funding

On a global scale, approximately one in every three adults will require rehabilitation over the course of their injury or illness (52). The causes span across a wide variety of pathologies, such as musculoskeletal, neurological, respiratory, cardiovascular and oncological disorders. As research in surgical and medical treatments continue to improve, so must PRM. However, as an example in the United State (i.e., NIH) in 2021, approximately $864 million was spent on rehabilitation overall (making it 64th) and approximately $220 million were spent on research in physical rehabilitation (making it 215th) out of the top 299 research disease topics (53). This ranking clearly shows that many funding agencies do not privilege clinical and translational research, while it is the last stone laid to confirm the efficacy of an intervention in real clinical practice.

## Developing Appropriate Quantification Strategies

In PRM, homogeneity and quantification of rehabilitative strategies is challenging. Indeed, as discussed in previous sections, the problem of heterogeneity is particularly present in rehabilitation. Rehabilitation can vary strongly from one therapist to another (experience, age, knowledge of therapists, choice of using “standard treatment” vs. “innovative treatment,” etc.), from one patient to another (comorbidities, patient's levels of cognitive deficit, patient willingness, etc.) and from one moment to another (emotional status, fatigue, etc.). In general, treatment and techniques need to be adapted to the level of physical and cognitive capacities of the patients (i.e., refers to the concept of *rehabilitation based on patients' deficits*). Therefore, quantifying rehabilitation techniques remains complicated. Yet, it is essential to use evidence based practice in all areas of medicine.

One solution to try to quantify the efficacy of rehabilitation is the use of questionnaires/scales. Parameters such as patient's walking capacities or balance can easily be done using validated tools.

However, clinicians and researchers are often confronted with challenges which are listed below (sections 6.1 to 6.3).

Of note, beside evaluating quantifying the efficacy of a specific intervention, the concept of System Impact Research aims to assess the impact of the health care system or cares pathways on patients in the context of rehabilitation (18). Such system is important to evaluate the impact of health policies on patients' health and the effectiveness of (novel) multidisciplinary rehabilitative pathways for specific diseases.

### The Use of Validated Tools

When different tools exist for a same purpose, researchers and clinicians would have to make a choice. One of the criteria of this choice should inevitably be the fact that the tool is/is not validated. However, it is not always clear what “validated” implies. Before using a scale or a questionnaire, it is recommended to verify the measurement properties of the tool. Ideally, the tool should have been tested on a similar population for its ability to measure what it claims to measure (i.e., its “validity”), its capacity to stay stable over time if the clinical status of patient is also stable (i.e., its “test-retest reliability”), its capacity to not be administrator-independent (i.e., its “inter-rater reliability”), its capacity to detect changes over time if the clinical status of the patient evolves, in a positive or negative way (i.e., its “sensitivity to change) and also, its homogeneity (i.e., its “internal consistency”) (54, 55). To know if a scale/questionnaire has been validated or not, it is necessary to identify scientific publications referring to this potential validation. When a clinician or a researcher in PRM plans to use a tool for a particular measurement, it is important to check if the tool has been validated specifically for this measurement. Indeed, it is likely that a tool has been validated analytically but not functionally or for one anatomical site and not for another. If the tool has not been validated for the specific target measurement, a validation study (i.e., analyzing clinometric properties of the tool in the target population) should be done prior using this tool for any clinical/research purpose.

### The Use of Tools That Have Been Translated and Validated in Their Own Language

Another important challenge is the availability of the tool/questionnaire in different languages. Indeed, a tool/questionnaire/scale is initially developed and validated in one unique language. If a French hospital desires to use a questionnaire that has been developed in English, and only been validated in English, the French researchers will have to first translate this tool then validate the translation to ensure that the translation has been correctly done. This process may be long and requires the use of a standardized methodology (56). For a scientific translation, it is necessary to follow different steps: first, the questionnaire should be translated from English to French independently by two bilingual translators, who have French as mother tongue. Second, the two translators should meet and agree on a first version of the translated questionnaire. Third, the translated French questionnaire needs to be back-translated independently by two bilingual translators, who this time have English as mother tongue and who are blinded to the original version. Four, a meeting should be organized with all the translators to agree on a second French version of the questionnaire, taking into account results from the back translations. Fifth and lastly, the pre-final translated version should be pre-tested on a sample of target population to ensure the translation is clear, understandable and free of language or grammatical errors. Once this last step is finalized, the version of the translated questionnaire may be considered as final and may be tested for its measurement properties.

### The Use of Generic vs. Specific Tools, Questionnaires or Scales

Clinicians and researchers should also be aware of the difference between generic scales and specific scales, the last one being specific to some populations and pathologies and being more sensitive to change. Because of the importance of specific evaluations depending on the clinical states of populations, many diseases-specific questionnaires have been developed in the last few years. In P&MR, the evolution of some pathologies could be very complex and may impact the choice of tool to use. Specific tools, more sensitive to change, may be very useful to evaluate, for example, a motor deficit at one moment and the short term impact of rehabilitation but could be less useful to use once the patient would have recover its motor capacities which is the case of the Medical Research Council (MRC) Scale for Muscle Strength (Lovett), widely used but not capturing fine improvement in motor function (57). Conversely, more generic tools, less sensitive to change, could lack the sensitivity to identify improvement following a particular rehabilitation but could be more useful to follow prospectively the evolution of a patient during their rehabilitation.

With better ability to research scientific literature, clinicians and researchers may be able to identify better tools to adequately assess outcomes of a rehabilitation procedure in a particular population. The use of appropriate, targeted and validated questionnaires/scales could allow for a better standardization in quantification of results in PRM practices.

### Analyses

In rehabilitation research, most assessment tools and outcomes measurements are not continuous but ordinal (e.g., Barthel Index, Health Assessment questionnaire), meaning that the distances between the raw score points are unequal and common statistical approaches (parametric tests) are invalid (58, 59). For such cases, statistical procedures (non-parametric test) are available for such ordinal outcomes (59).

As most interventional studies in rehabilitation and health in general wish to calculate a change score, or use values from ordinal scales in procedures such as ANOVA and regression, then the challenge is to provide a transformation of such scale to the interval level. In this context, Rasch proposed the Rasch analysis, which is a psychometric technique allowing researchers to construct alternative forms of measurement instruments (60). For a detail description of the procedure see (61).

Besides the Rasch analysis, depending on the data acquired (categorical, ordinal, continuous) and their distributions, specific statistical tests could be performed (e.g., parametric or non-parametric statistics). Several tools have been developped to help researcher finding the appropriate test based on the type of data they collected (62, 63).

## Ethical Challenges

One important challenge in PRM is the ethical aspects of conducting trials in patients who might suffer from cognitive deficits. Based on the declaration of Helsinki, guidelines have been developed to ensure that consent to participate in a research protocol is done based on the patient's best interest. To comply with this regulation, three conditions must be met, namely: *patient capacity* (the patient's ability to understand the nature of the research, as well as its risks and benefits, in order to make an informed decision), *voluntariness* (freedom from undue coercion, be it deliberate or unintended) and *disclosure* (the provision of all information necessary for the potential subject to assist them in the decision-making process).

However, a significant proportion of patients in rehabilitation medicine have cognitive deficits, of varying severity, that can compromise the patient's ability to 1. understand the ins and outs of a research protocol, and 2. be able to make a lucid decision about his or her interest in taking part in a research protocol. In this context, the researcher must be vigilant in ensuring that the subject's participation is truly informed and voluntary. If this is not the case, the intervention of a relative and/or the patient's legal representative will be necessary.

Since rehabilitation care is often lengthy, spanning several years, another ethical consideration is the number of protocols a patient may participate in. Indeed, it is possible that some patients may be recruited for multiple studies throughout the course of their rehabilitation. According to the ethical principle of justice, which examines the distribution of the costs and benefits of living in a society, no group should bear a disproportionate burden of participation in research. For patients whose stay exceeds a certain length of time, it might be reasonable to put guidelines in place so that these patients are only invited to participate in a certain number of research projects, perhaps one per year or one every two years, to ensure that they do not bear an excessive burden in this area.

## Concluding Remarks

To conclude, we here summarize the key points discussed in the previous sections.

Regarding the study design, RCT are the gold standard, which provide the strongest scientific evidence necessary to promote evidence based practice. However, alternative such as observational BCTs can be choosen if the aim is to assess the effectiveness of a program or healthcare pathway (as opposed to the efficacy of a specific intervention). Other studies can also be conducted and bring useful information, such as cohort studies in term of risk factors of developing a disease or exploratory uncontrolled open-label study, which can bring important insights on the possible efficacy of an intervention as well as its safety and feasibility. In parallel, pragmatic trials (a type of RCT) are designed to evaluate the effectiveness of an intervention in real-life routine practice, and are well suited for PRM. Importantly, no matter which design is chosen, it is crucial to follow the available checklists and guidelines (e.g., CONSORT checklist for RCT).

When performing a RCT, choosing an appropriate controlled condition can be complex, as is ensuring adequate blinding. The use of active placebos may be an elegant solution, however, it cannot be applied to all interventions. If the blinding of both the participant and the evaluator is questionable in an RCT, this must be recognized as a limitation since performance bias cannot be excluded.

In some cases, is also important to quantify the amount of therapy provided which may vary among participants due to population heterogeneity. A detailed procedure and systematic reporting of the intervention are therefore critical. To avoid biases linked to population heterogeneity, applying stringent inclusion criteria is recommended; however, it also decreases the external validity of the study and may leads to smaller sample sizes and lower statistical power.

To overcome the issue of sample size and increase the external validity and thus enhance the changes for a successful clinical translation, large multicenter pragmatic trials, despite the logistical challenges, should be implemented. Validated scales and common data elements should be used to ensure an exhaustive reporting.

Regarding recruitment and adherence, while they represent an extra challenge in PRM given the length of most intervention, the communication and reliability between the clinician/research and the patient is key. Similarly, all research undertaken must follow the declaration of Helsinki to ensure that the research protocol is done based on the patient's best interest.

To sum up, developing robust experiment design in PRM might be challenging. However, many solutions exist to tackle potential biases. When these biases are unresolvable, clear and honest reporting of the limitations is essential. Therefore, a thorough knowledge of these challenges is crucial.

## Author Contributions

All authors listed have made a substantial, direct, and intellectual contribution to the work and approved it for publication.

## Conflict of Interest

The authors declare that the research was conducted in the absence of any commercial or financial relationships that could be construed as a potential conflict of interest.

## Publisher's Note

All claims expressed in this article are solely those of the authors and do not necessarily represent those of their affiliated organizations, or those of the publisher, the editors and the reviewers. Any product that may be evaluated in this article, or claim that may be made by its manufacturer, is not guaranteed or endorsed by the publisher.
